# Effect of beta blockers on the incidence of atrial fibrillation in elderly patients after abdominal surgery

**DOI:** 10.1186/1471-2482-13-S1-A32

**Published:** 2013-09-16

**Authors:** Gennaro Pagano, Dario Leosco, Nicola Ferrara, Nicola Rocco, Corrado Rispoli, Loredana Iannone, Serena Testa, Rita Compagna, Antonello Accurso, Bruno Amato

**Affiliations:** 1 Department of Translational Medical Sciences, Federico II University of Naples, Naples, Italy; 2 “Salvatore Maugeri” Foundation, IRCCS Scientific Institute of Telese Terme, Benevento, Italy; 3 Department of Biomedical, Surgical and Dental Sciences, University of Milan, Milan, Italy; 4 Department of General Surgery - ASL NA1, Cardinale Ascalesi Hospital, Naples, Italy; 5 Department of General Surgery, Fatebenefratelli Hospital, Benevento, Italy; 6 Department of General, Geriatric, Oncologic Surgery and Advanced Technologies, Federico II University of Naples, Naples, Italy

## Aim of the study

To determine whether treatment with Beta Blockers during abdominal surgery would reduce the incidence of post-operative Atrial Fibrillation (AF).

## Background

Post operative AF is a common problem in patients following surgery. The incidence of such arrhythmia has been reported to range from 20–35% and increase to the age and to presence of comorbidities [1-3]. Also, AF is related to many complications and it is still debated what is the best interventions, especially in the elderly [4]. Sympathetic nervous system activation is strictly related to surgery stress [5] and treatment with Beta Blockers may reduce this overactivation that predispose to AF occurence [6-10].

## Methods

A pilot study was conducted with 30 elderly patients (age > 65 years) who underwent abdominal surgery (right emicolectomy, sigmoidectomy and anterior rectal resection). They were randomized to control (ctr) (n = 14) and Beta Blockers (beta) (n = 16) groups. Before surgery all elderly patients have been taking Beta Blockers (for hypertension or heart failure). During and after surgery only the beta group received the medication. Both groups were well matched and had the same demographic and clinical characteristics.

## Results

After surgery 5 patients of ctr group and in 1 patients of beta group developed AF (p < 0.001). All patients with AF occurrence were >75 years old.

## Conclusions

Beta Blockers could not be stopped during abdominal surgery in elderly patients (with hypertension or heart failure) because significantly reduce the incidence of post-operative AF. Moreover, the link between the older age and AF occurrence is confirmed also in this population.
